# Hyperglycemia is associated with poor survival in patients with brain metastases treated with radiotherapy

**DOI:** 10.1007/s00066-025-02414-y

**Published:** 2025-06-16

**Authors:** Soniya Poudyal, Friederike Rothe, Seong Jeong, Nils Gleim, Peter Hambsch, Franziska Nägler, Kirsten Papsdorf, Thomas Kuhnt, Alonso Barrantes-Freer, Erdem Güresir, Sabine Klagges, Nils Henrik Nicolay, Clemens Seidel

**Affiliations:** 1 Department of Radiation Oncology, University of Leipzig Medical Center, Leipzig, Germany; 2 Clinical Cancer Registry Leipzig, Leipzig, Germany; 3 University of Leipzig Medical Center, Paul-Flechsig-Institute of Neuropathology, Leipzig, Germany; 4 Department of Neurosurgery, University of Leipzig Medical Center, Leipzig, Germany; 5 Comprehensive Cancer Center Central Germany, Leipzig, Germany; 6 Stephanstraße 9a, 04103 Leipzig, Germany

**Keywords:** Brain metastasis, Radiotherapy, Hyperglycemia, Diabetes mellitus, Dexamethasone

## Abstract

**Purpose:**

Diabetes mellitus (DM) is a negative prognostic factor in patients with brain metastases (BM). It is unknown whether a direct impact of serum glucose levels on survival exists. We aimed to detect a potential association of serum glucose levels before and during radiotherapy with survival in patients with BM.

**Methods:**

Patients were included in this retrospective exploratory analysis if at least three fasting and non-fasting serum glucose test results before or during treatment were available. Survival was analyzed with uni- and multivariate Cox regression concerning an association with fasting and maximum glucose levels and regarding potentially confounding dexamethasone intake.

**Results:**

A total of 62 patients with BM (15 with and 47 without DM) were included. Patients with a mean fasting glucose of more than 7.8 mmol/l (upper quartile) showed significantly shorter survival compared to patients of the lower three quartiles (hazard ratio [HR] = 2.05, *p* = 0.021). Further, maximum blood glucose levels of > 12.0 mmol/l (upper quartile) were associated with shorter survival (HR = 1.95, *p* = 0.035). In the subset of patients without DM, a trend toward worse survival in patients with higher fasting glucose levels was observed (HR = 2.54, *p* = 0.099). The negative association of high maximum glucose levels with survival persisted in multivariate analysis independently of steroid administration.

**Conclusion:**

Strong elevations of fasting and maximum serum glucose levels were associated with a worse prognosis in patients with BM with and without DM. This observation warrants further analysis in larger cohorts and has potential implications for clinical practice.

**Supplementary Information:**

The online version of this article (10.1007/s00066-025-02414-y) contains supplementary material, which is available to authorized users.

## Introduction

The occurrence of brain metastases (BM) is a prevalent and severe complication of cancer, impacting approximately 30% of patients diagnosed with malignant tumors [1, 2]. As life expectancy continues to increase and cancer survivorship improves, the incidence of BM is expected to increase [3, 4]. Despite the emergence of more effective therapeutic options, patients with BM often retain a poor prognosis with short median survival [2–6]. Numerous factors, such as age, performance status, or frailty, can potentially affect prognosis [7–10].

In addition, diabetes mellitus (DM) is known to have detrimental effects in cancer patients. Evidence suggests that cancer patients with DM have higher cancer-related mortality and that patients with DM are at an increased risk of other morbidity and mortality [11–14]. Diabetes mellitus can impact cancer patients’ outcomes via long-term effects that are also thought to influence tumor growth [12, 15].

Increased serum glucose levels—also known as hyperglycemia—constitute an important feature of DM [16]. Hyperglycemia is thought to play a key role in tumor progression by reprogramming glucose metabolism and stimulating cancer-associated inflammation, induction of hypoxia, and resistance to radiotherapy [17–20]. Hyperglycemia during treatment of primary brain tumors has been linked to poorer patient outcomes [21–23], but the influence of serum glucose levels on the outcomes of patients with brain metastases has not yet been examined.

The aim of this exploratory study was to evaluate the association of serum glucose levels with overall survival (OS) in patients with and without diagnosed DM. We further wanted to evaluate whether antidiabetic treatment and corticosteroid therapy may influence the prognosis in the context of hyperglycemia.

## Materials and methods

This research study was conducted in accordance with the Declaration of Helsinki and received approval from the Ethics Committee of the University of Leipzig (date: 03.08.2021, no. 332/21-ek). All patients provided informed consent for the anonymous scientific use of their clinical data. The study cohort consisted of patients who had undergone radiotherapy at the Department of Radiation Oncology, University Hospital of Leipzig, Germany, between 2004 and 2015 who had been diagnosed with BM from a solid primary tumor and were aged 18 years or older. We selected consecutive patients with accessible pretherapeutic cerebral MRI scans and comprehensive clinical records. The choice of treatment, i.e., whether it was stereotactic radiotherapy (SRT) or whole-brain radiotherapy (WBRT), depended on factors such as the number of metastases and the patient’s overall health. A standardized approach was used to record patient characteristics. These included age at the time of primary BM diagnosis, type of primary tumor, Karnofsky performance status (KPS), number of metastases, systemic tumor control, number of prior systemic treatment lines, and the presence or absence of DM and other cardiovascular comorbidities, which were recorded prior to initiating treatment for BM. Additionally, other relevant patient data, including morning fasting serum glucose levels, serum glucose levels at different times of the day, treatment for patients with DM before and during radiotherapy, and co-administered corticosteroid therapy, were extracted retrospectively from existing electronic patient records. Serum glucose levels were collected within 3 months before and during radiotherapy. For inclusion in the study, at least three fasting glucose and at least three non-fasting glucose levels had to be available per patient. Individually, from fasting glucose levels, mean fasting glucose levels, and non-fasting glucose levels, maximum glucose levels were calculated. Treatment for patients previously diagnosed with type 2 DM, either with oral medication or insulin, was also obtained. Survival data were obtained from the local cancer registry, and data analysis was performed using Microsoft Excel 2016 (Microsoft Corporation, Albuquerque, NM, USA) and SPSS version 28.0.1.1 (IBM Corp., Armonk, NY, USA). Univariate and multivariate survival analysis was conducted using Kaplan–Meier curves and Cox regression analysis.

## Results

### Patient characteristics

Out of 203 patients with available survival data, 62 had sufficient available serum glucose values and were thus included in the study. Patient age at the time of brain metastasis diagnosis ranged from 30 to 84 years (median 60.35 years, mean 61.75 years). At BM diagnosis, patients presented with a median KPS of 70% (range 30–100%). The most prevalent primary tumor was non-small cell lung cancer (NSCLC), which accounted for 25 of the 62 patients (40.3%). Primary tumor types also included melanoma (14/62, 22.6%), breast cancer (7/62, 11.3%), small cell lung cancer (SCLC; 7/62, 11.3%), renal cell carcinoma (RCC; 2/62, 3.2%), and various other cancers (7/62, 11.3%; Table 1). Regarding baseline characteristics, 36/62 patients (58.1%) were male, and 26/62 (41.9%) were female. Peripheral arterial occlusive disease or coronary heart disease (PAD/CHD) was present in 4/62 (6.5%) patients. Arterial hypertension was documented in 34/62 patients (54.8%). Hypercholesterolemia was noted in 4/62 (6.5%) patients, while a positive smoking history was reported in 23/62 (37.1%) patients. While 38.8% of patients (24/62) were diagnosed with 1–3 BM, 61.3% (38/62) had more than three BM. Treatment of these BM included stereotactic radiotherapy (SRT) for 11/62 patients (17.7%), whole-brain radiotherapy (WBRT) with additional SRT for 25/62 patients (40.3%), and WBRT for 26/62 patients (41.9%).

**Table 1:** Patient characteristics

Characteristic	Group	Patients, *n* (%)
Age	< 70 years	46 (74.2%)
	≥ 70 years	16 (25.8%)
KPS	≥ 70	36 (58.1%)
	< 70	22 (35.5%)
	Missing data	4 (6.5%)
No. of brain metastases	≥ 3	38 (61.3%)
	< 3	24 (38.7%)
Tumor type	NSCLC	25 (40.3%)
	SCLC	7 (11.3%)
	Breast cancer	7 (11.3%)
	Melanoma	14 (22.6%)
	RCC	2 (3.2%)
	Other	7 (11.3%)
Radiotherapy technique	SRT	9 (14.5%)
	WBRT + SRT	26 (41.9%)
	WBRT only	27 (43.51%)
Diabetes mellitus	Yes	15 (24.2%)
	No	47 (75.8%)
	Missing data	0
DM therapy	Oral antidiabetic/insulin	9 (14.5%)
	No treatment	49 (79%)
	Missing data	4 (6.5%)
Dexamethasone use	Yes	26 (41.9%)
	No	36 (58.1%)
	Missing data	0
Previous systemic treatment lines	0	23 (37.1%)
	≥ 1	35 (56.5%)
	Missing	4 (6.4%)

*KPS* Karnofsky performance status, *NSCLC* non-small cell lung cancer, *SCLC* small cell lung cancer, *RCC* renal cell carcinoma, *SRT* stereotactic radiotherapy, *WBRT* whole-brain radiotherapy, *DM* diabetes mellitus

Diabetes mellitus had been diagnosed prior to treatment in 15/62 (24.2%) patients. With regards to systemic treatment, 35/62 patients (56.5%) had received at least one systemic treatment before diagnosis of BM, and 11/62 (17.7%) had received at least two systemic treatments before their BM diagnosis.

Nine patients were treated with either insulin or oral antidiabetic medication. Among the 62 patients, 26 (41.9%) received dexamethasone, while 36 (58.1%) did not receive corticosteroids.

**Fig. 1:**
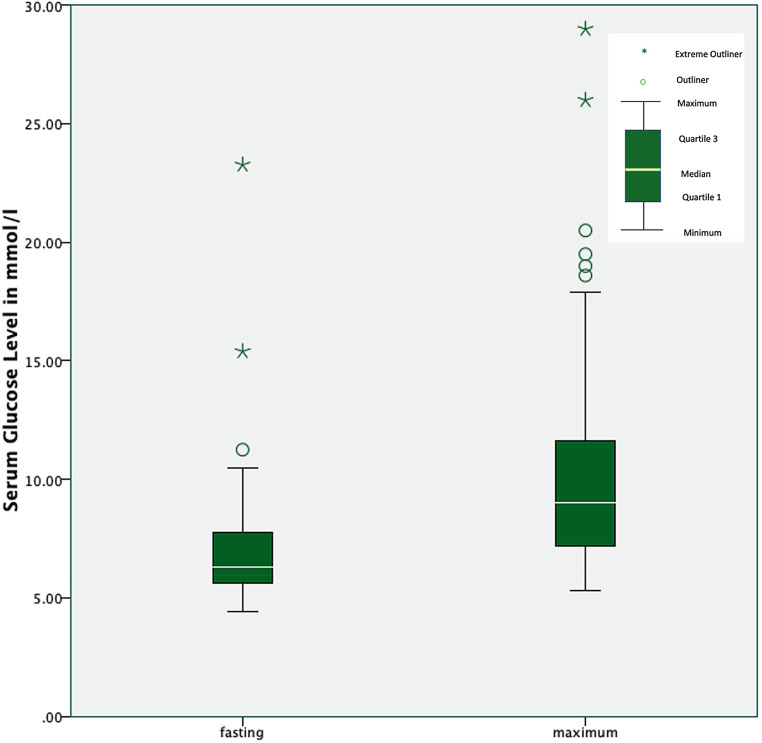
Box and whisker plots representing patients’ mean fasting and mean maximum serum glucose levels. The median is represented by a white line located in the middle of the box. The top and bottom of the box represent the 75th and 25th percentiles, respectively, and the ends of the whiskers represent the 75th (or 25th) percentile ±1.5 times the interquartile range. Green circles represent mild outliers, and green stars represents extreme outliers

The patient group demonstrated a median fasting serum glucose level of 6.4 mmol/l (range 4.50–23.27 mmol/l). The upper serum glucose limits of the first (25%), second (50%), and third (75%) quartiles were 5.6 mmol/l, 6.4 mmol/l, and 7.82 mmol/l, respectively. The median maximum glucose level was 9.0 mmol/l (range 5.3–29.0 mmol/l), and the corresponding upper quartile limits (25%, 50%, and 75%) were 7.4 mmol/l, 9.0 mmol/l, and 12.02 mmol/l, respectively (Fig. 1).

### Patients with high fasting and maximum glucose levels exhibit decreased overall survival

**Fig. 2:**
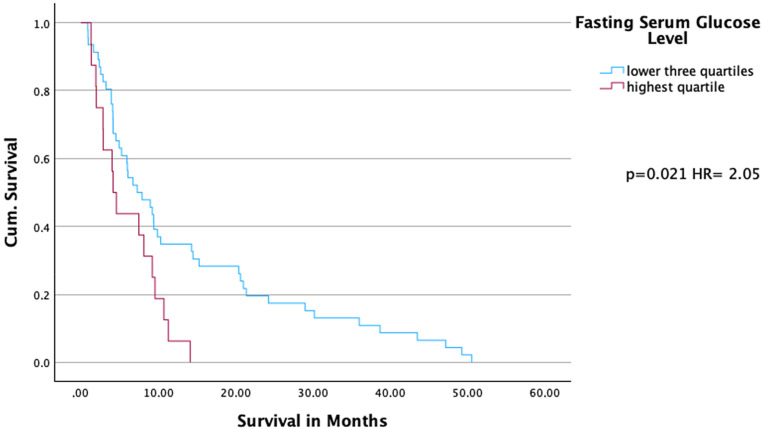
Cumulative survival of patients with fasting serum glucose levels in the highest and lower three quartiles. Kaplan–Meier curves with hazard ratio (HR) and the *p*-value of Cox regression analysis are shown

Survival times of all patients were available. The median survival time was 7.01 months (range 0.89–50.53), with a median follow-up of also 7.01 months. Investigating the impact of fasting serum glucose on survival by distinguishing between patients in the highest quartile and those in the lower three quartiles showed significantly shorter survival in the highest quartile compared to the patients in the lower three quartiles (hazard ratio [HR] = 2.05, 95% confidence interval [CI]: [1.12, 3.76], *p* = 0.021). Median overall survival in the highest quartile was 4.2 months, compared to 7.3 months in the lower three quartiles. The upper quartile exhibited lower 1‑ (0.063 vs. 0.35) and 2‑year (0.00 vs. 0.174) overall survival rates (Fig. 2).

Furthermore, fasting serum glucose levels of > 7.82 mmol/l (highest quartile) were also significantly associated with shorter survival (HR = 2.83, 95% CI: [1.14, 7.05], *p* = 0.025) compared to the lowest quartile. Median overall survival was reduced in the highest quartile (4.17 months) compared to the lowest quartile (8.9 months). The 1‑year survival rate was 0.063 vs. 0.385 (Supplement Fig. 1).

**Fig. 3:**
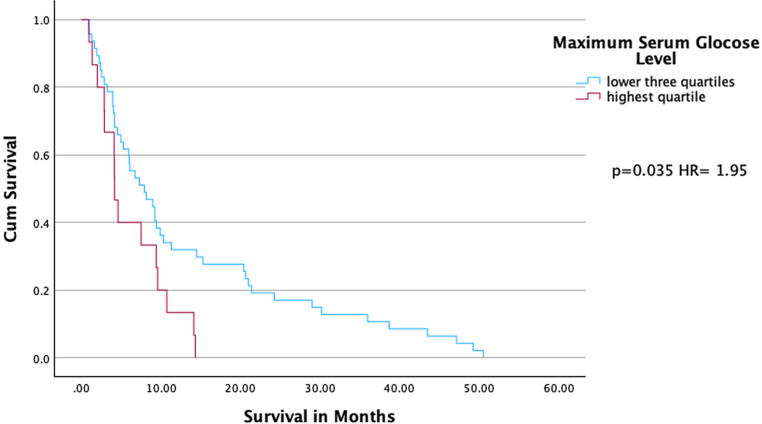
Cumulative survival of patients with maximum serum glucose levels in highest and lower three quartiles. Kaplan–Meier curves with hazard ratio (HR) and the *p*-value of Cox regression analysis are shown

Similarly, patients with maximum serum glucose levels in the highest quartile showed significantly shorter survival compared to patients of the lower three quartiles (HR = 1.95, 95% CI: [1.05, 3.60], *p* = 0.035). Median overall survival was reduced in the upper quartile (4.2 months) compared to the lower three quartiles (7.9 months). The highest quartile exhibited lower 1‑ (0.133 vs. 0.319) and 2‑year (0.0 vs. 0.17) survival rates compared to the lower three quartiles (Fig. 3).

Further, maximum serum glucose levels of 12.02 mmol/l (highest quartile) showed a trend toward shorter survival (HR = 2.056, *p* = 0.095) compared to the lowest quartile. Median survival was reduced in the upper quartile (4.1 months) compared to the lowest quartile (4.9 months). The highest quartile exhibited a lower 1‑year survival rate (0.133 vs. 0.33) compared to the lowest quartile (Supplement Fig. 2).

The median fasting serum glucose level in this cohort of patients was 6.4 mmol/l, while the median for the maximum serum glucose level was 9.0 mmol/l. When median glucose values served as the cutoff for survival analysis, no significant differences were observed for a) fasting serum glucose level </≥ 6.4 mmol/l (*p* = 0.293) or b) maximum serum glucose level </≥ 9.0 mmol/l (*p* = 0.275; Supplement Fig. 3).

In a restricted analysis encompassing *n* = 47 patients with available survival data and without diagnosed DM, several trends were observed in interquartile comparisons concerning both fasting and maximum serum glucose levels. Between the highest quartile (7/47) of fasting serum glucose levels and the lower three quartiles (40/47), no significant effect on survival was noted (HR = 1.79, *p* = 0.178). In the comparison of the subset of 19 patients from the highest (7/19) and lowest (12/19) quartiles, a trend towards worse survival in patients with the highest fasting glucose quartile was observed (HR = 2.54, *p* = 0.099; Supplement Fig. 4). Similarly, when analyzing maximum serum glucose levels in interquartile comparisons, trends toward deteriorated survival with higher glucose levels that did not reach significance were observed (Supplement Fig. 5).

Concerning potential effects of antidiabetic treatment, patients with (oral ± insulin; 10/62) and without treatment for DM did not show a difference in survival, while the OS of patients without DM was significantly longer (HR = 0.73, 95% CI: [0.54, 0.98], *p* = 0.039; Supplement Fig. 6).

In the univariate Cox regression analysis, patient age ≥ 70 years showed significant detrimental effects in terms of survival (HR = 1.96, 95% CI: [1.29, 3.00], *p* = 0.026). Further, if BM occurred after at least one line of prior systemic therapy, survival was significantly shorter (HR = 2.03, 95% CI: [1.15, 3.56], *p* = 0.015). However, between patients with ≥ 2 systemic treatment lines (*n* = 11) and patients with 0–1 systemic treatment lines before BM diagnosis (*n* = 47), no significant survival difference was observed (HR = 0.69, *p* = 0.27).

A KPS < 70 and tumor histology were not associated with reduced survival (KPS < 70: HR = 1.00, *p* = 0.139; tumor histology: HR = 1.01, *p* = 0.94). The number of BM, radiotherapy technique, and corticosteroid use were not associated with differences in survival time (≥ 3 BM: HR = 1.06, *p* = 0.82; radiotherapy technique: HR = 1.47, *p* = 0.319; corticosteroid use: HR = 1.14, *p* = 0.611). Similarly, gender, arterial hypertension, PAD/CHD, smoking, and hypercholesterolemia showed no significant association with survival time (gender: HR = 1.41, *p* = 0.20; hypercholesterolemia: HR = 1.83, *p* = 0.25; smoking: HR = 1.14, *p* = 0.624; arterial hypertension: HR = 1.11, *p* = 0.68; PAD/CHD: HR = 0.80, *p* = 0.670; Supplement Table 1).

### Association of high serum glucose level and reduced survival persists in multivariate analysis

Multivariate Cox regression analysis was performed, including fasting/maximum serum glucose level (highest vs. lower quartiles) and established prognostic factors, i.e., age (< 70, ≥ 70 years), KPS score (< 70, ≥ 70), systemic treatments (no/yes), number of BM (≤ 3, > 3), primary tumor type, and radiotherapy concept. Other factors such as arterial hypertension, PAD/CHD, hypercholesterolemia, and smoking were not included in multivariate analysis due to their insignificant effect in univariate analysis.

**Fig. 4:**
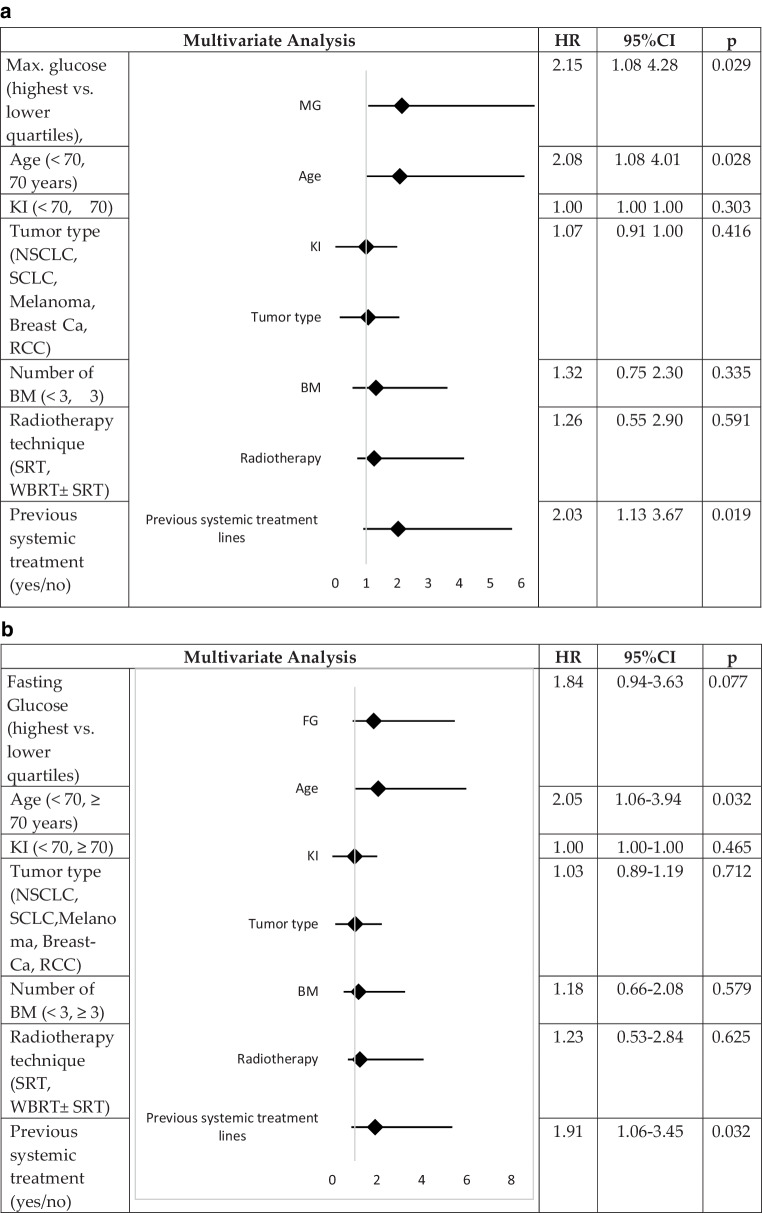
Multivariate analysis of survival for maximum serum glucose level (**a**) and fasting serum glucose level (**b**). *KI* Karnofsky index, *NSCLC* non-small cell lung cancer, *SCLC* small cell lung cancer, *RCC* renal cell carcinoma, *BM* brain metastases, *SRT* stereotactic radiotherapy, *WBRT* whole-brain radiotherapy

In this analysis, high maximum serum glucose levels (HR = 2.15, 95% CI: [1.08, 4.28], *p* = 0.029), age ≥ 70 years (HR = 2.08, 95% CI: [1.08, 4.01], *p* = 0.028), and prior systemic treatment (HR = 2.03, 95% CI: [1.13, 3.67], *p* = 0.019) remained independently related to shorter survival. No effects were identified for the number of BM, KPS score, tumor type, and radiotherapy technique (Fig. 4a).

For fasting glucose, a trend was observed toward shorter survival with higher serum glucose levels (HR = 1.84, 95% CI: [0.94, 3.63], *p* = 0.077).

Further, age (≥ 70 years) was independently associated with poorer survival (HR = 2.05, 95% CI: [1.06, 3.94], *p* = 0.032), as was prior systemic treatment (HR = 1.91, 95% CI: [1.06, 3.45], *p* = 0.032). No effect was noted for the number of BM, KPS score, primary tumor type, or radiotherapy technique (Fig. 4b).

In an additional multivariate analysis, we investigated a prognostic effect of a) fasting and b) maximum serum glucose levels categorized into the upper quartile versus the lower three quartiles, with dexamethasone use as a confounding factor.

For fasting serum glucose, elevated glucose levels in the upper quartile were associated with a significantly elevated hazard ratio (HR = 2.15, 95% CI: [1.15, 4.01], *p* = 0.016), while dexamethasone usage was not (HR = 1.26, *p* = 0.385).

Similarly, in the analysis of maximum serum glucose levels, high maximum glucose levels were associated with significantly poorer survival (HR 2.13, 95% CI: [1.12, 4.05], *p* = 0.022), while use of dexamethasone was not (HR = 1.319, *p* = 0.311).

## Discussion

In this study, we investigated the potential influence of hyperglycemia on the survival of patients with BM. The metabolic determinants that appear to have an impact on survival in malignancies in general are subject of ongoing discussions [24, 25]. Already in 1930, Otto Warburg was the first to describe that cancer cells obtain their energy mainly by anaerobic metabolization of glucose despite sufficient oxygen supply [26, 27]. Several studies also suggest an association between hyperglycemia and tumor growth, progression, recurrence, mortality, and decreased survival in various types of tumors, including colorectal cancer, liver cancer, pancreatic cancer, breast cancer, and lung cancer [14, 28–34].

In our cohort of patients with BM and frequently measured serum glucose, patients with higher fasting and maximum serum glucose levels had significantly worse OS. Also, in patients without diagnosed DM, a trend toward prognostic deterioration was discernible. While data are lacking on this topic in patients with BM, there is relevant clinical evidence from malignant glioma. McGirth and colleagues found that a group of 367 patients with malignant astrocytoma (grade 3 and 4) had reduced survival rates when experiencing persistent hyperglycemia. Blood glucose levels higher than 180 mg/dl were defined as hyperglycemic. Of the patients who underwent surgery, 19% experienced isolated hyperglycemia, and 8% had persistent hyperglycemia in the first 3 months after surgery. Patients who experienced persistent hyperglycemia had a median survival of 5 months compared to patients with normoglycemia who survived for 11 months [35].

Interestingly, in our analysis, comparisons focusing on the highest quartile of fasting or maximum serum glucose levels showed worse survival in this group, while comparisons focusing on serum glucose above and below median did not show a significant difference in survival. Potentially, mild hyperglycemia might be less detrimental. However, Adeberg and colleagues found in a group of 262 patients with malignant glioma that both persistent mild hyperglycemia (HR = 2.23, *p* < 0.001) and excessive hyperglycemia (HR = 2.51, *p* < 0.001) were independently linked to reduced overall survival rates when accounting for the covariate of corticosteroid therapy [22]. Also, in our analysis, the negative prognostic impact of maximum glucose level persisted in multivariate analysis independently of age, KPS, and the use of dexamethasone. Dexamethasone use is known to be associated with a reduced prognosis in malignant brain tumors [36].

Regarding treatment of DM, patients with and without (oral ± insulin) DM treatment did not show differing survival, while the OS of patients without DM was significantly longer. With a very limited sample size, this observation indicates that patients with high serum glucose levels experience worse survival outcomes despite treatment. Most likely, the use of antidiabetic treatment is associated with the presence of advanced DM, and conclusions concerning effects of antidiabetic treatment on OS in patients with BM cannot be drawn from our analysis.

Many consequences of DM promote tumor metabolism. Hyperglycemia, hyperinsulinemia, and a diabetes-associated chronic inflammatory state appear to be associated with elevated cancer mortality [32, 37]. In a large prospective cohort study, hyperinsulinemia, even without manifest DM, was associated with increased cancer mortality [38].

Compared to nonmalignant cells, the major needs for proliferation of malignant cells are satisfied by glucose only, and deregulated increasing proliferation is facilitated by high glucose levels [39–41]. A certain critical threshold for growth promotion through hyperglycemia appears, in our opinion, less likely than a continuously increasing negative effect of increasing glucose levels. However, deeper knowledge concerning this issue is important.

Further, and in a more regulatory sense, hyperglycemia promotes tumor progression by reprogramming glucose metabolism, by major malignant molecular alterations of peritumoral inflammation, and by local immunosuppression, contributing to poorer outcomes of tumor patients [17–20, 42–44].

**Fig. 5:**
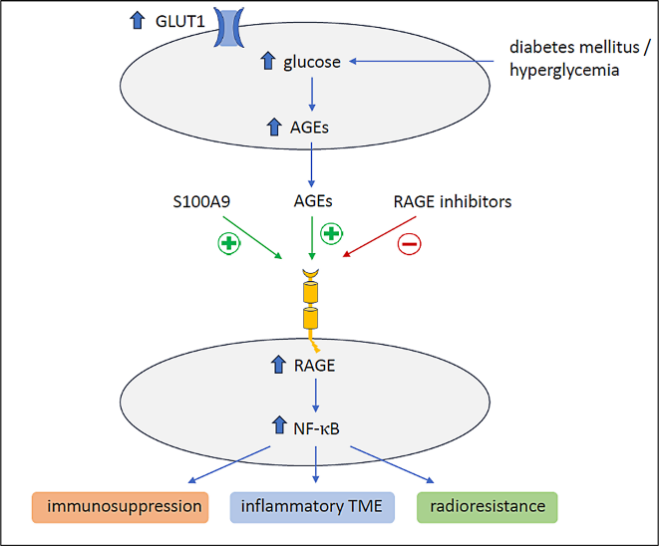
Role of receptor for advanced glycation end products (*RAGE*) activation in bone metastases (BM) and diabetes mellitus; RAGE can be activated by several mechanisms including hyperglycemia, and, via NF-κB, contributes to immunosuppression as well as an inflammatory tumor microenvironment and, eventually, radioresistance of BM. RAGE activation can be reversed by specific receptor inhibitors. *AGE* advanced glycation end products

Another important regulator in the interplay between diabetes mellitus, tumor cells, and treatment is the receptor for advanced glycation end products (RAGE). The RAGE receptor is activated as a consequence of an increased glycolysis that enhances the nonenzymatic glycation of proteins, leading to the formation of advanced glycation end products (AGEs) [45]. These AGEs, particularly N‑carboxymethyllysine [CML]-modified proteins, were the first identified RAGE ligands [45]. Overexpression and activation of RAGE are able to continuously fuel an inflammatory milieu in the tumor microenvironment [46, 47]. Interestingly, the overactivation of RAGE as part of the S100A9-RAGE-NF-κB-JunB pathway was discovered as a mechanism for the radioresistance of brain metastasis as well [48]. Brain metastatic cancer cells from different primary tumors were found to highly express S100A9 in the brain microenvironment, mediating resistance to radiotherapy via the downstream activation of RAGE and NF-κB. In patients with lung cancer, breast cancer, or melanoma, S100A9 expression in brain metastases negatively correlated with the benefits of radiotherapy. Genetic or pharmacological targeting of S100A9 via a blood–brain-barrier-permeable inhibitor of its receptor (RAGE) sensitized brain metastases to irradiation in experimental models of brain metastasis as well as in patient-derived organotypic cultures [48]. Diabetes mellitus/hyperglycemia might confer radioresistance via RAGE activation—potentially independently of S100A9 (Fig. 5).

As a further molecular crosslink between altered glucose metabolism and treatment of BM, a recent multiplex immunofluorescence study of resected BM samples from 33 patients treated with radiotherapy and ipilimumab for BM of melanoma found strong upregulation of the glucose transporter GLUT 1 in BM to be associated with a prognostic detriment in these patients [49].

In addition, it can be speculated that hyperglycemia is particularly relevant in BM or other malignant brain tumors due to constant high perfusion.

To the best of our knowledge, this study is the first to assess the association between hyperglycemia and overall survival via uni- and multivariate analysis in patients with BM.

It remains unclear whether the observed findings reflect a direct effect of high glucose levels and tumor growth on survival or rather indirect negative complications in hyperglycemic patients. Further, the limitations of the retrospective study character need to be acknowledged. We aimed to analyze patients with several available blood tests only, which reduced patient numbers and introduced some selection bias but should have led to a more robust dataset.

Further research, including prospective investigations, is warranted to elucidate the clinical impact of hyperglycemia and its treatment during radiotherapy for BM in more detail. However, it can be postulated from the existing data in malignant glioma and from our BM dataset that treating physicians should monitor and adequately treat elevated serum glucose levels in this fragile patient population.

## Supplementary Information

Figure series including additional cummulative survival comparisons and univariate survival analysis

## Data Availability

The datasets generated during and/or analyzed during the current study are available from the corresponding author upon reasonable request.
