# Supplementary material for: Hyperglycemia is associated with poor survival in patients with brain metastases treated with radiotherapy
Source: Strahlenther Onkol. 2025 Jun 16;201(9):920–9. doi: 10.1007/s00066-025-02414-y (PMC12373678; doi:10.1007/s00066-025-02414-y)
Supplement: Supplementary file 1 — Figure series including additional cummulative survival comparisons and univariate survival analysis [file 66_2025_2414_MOESM1_ESM.docx]

**Supplementary Materials**

Figure 1: Cumulative survival of patients with fasting serum glucose level in highest and lowest quartile. Kaplan-Meier curves with Hazard ratio (HR) and p-value of Cox-Regression analysis are shown.

**
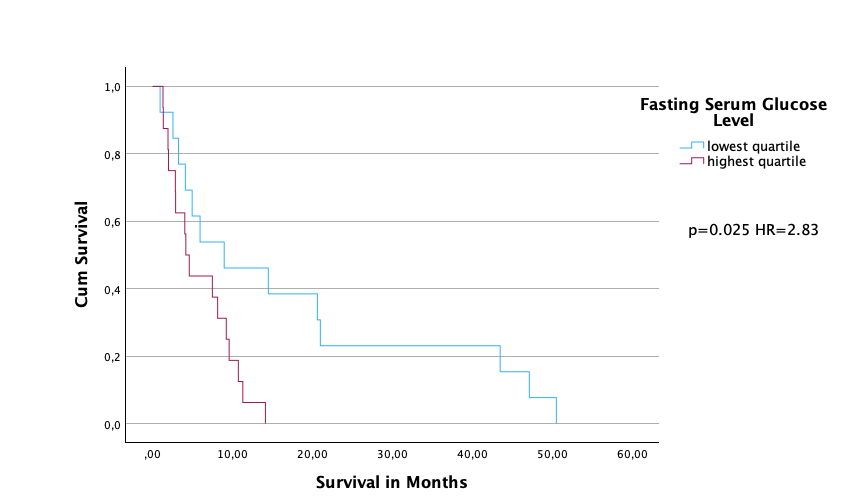
**

Figure 2: Cumulative survival of patients with maximum serum glucose level in highest and lowest quartile. Kaplan-Meier curves with Hazard ratio (HR) and p-value of Cox-Regression analysis are shown


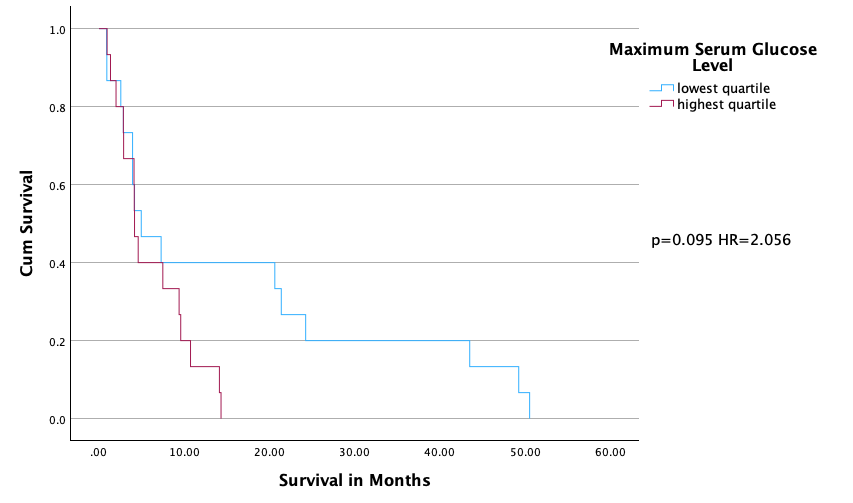


Figure 3A: Survival of patients with fasting glucose level under and over the median.


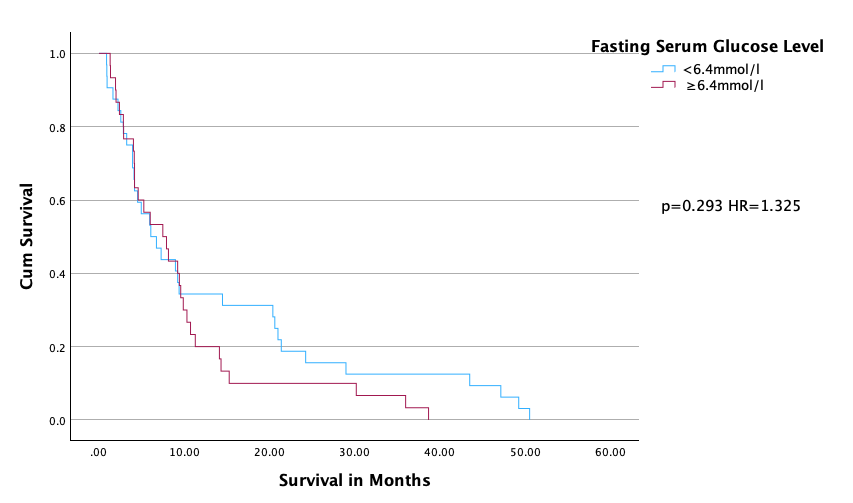


Figure 3B: Survival of patients with maximum glucose level under and over the median


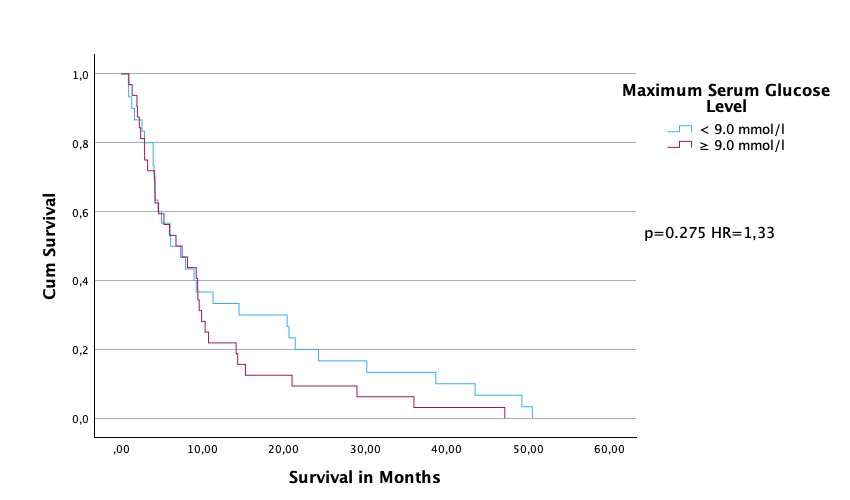


Figure 4A: Survival curves for patients without DM with fasting serum glucose level in highest and lower three quartiles


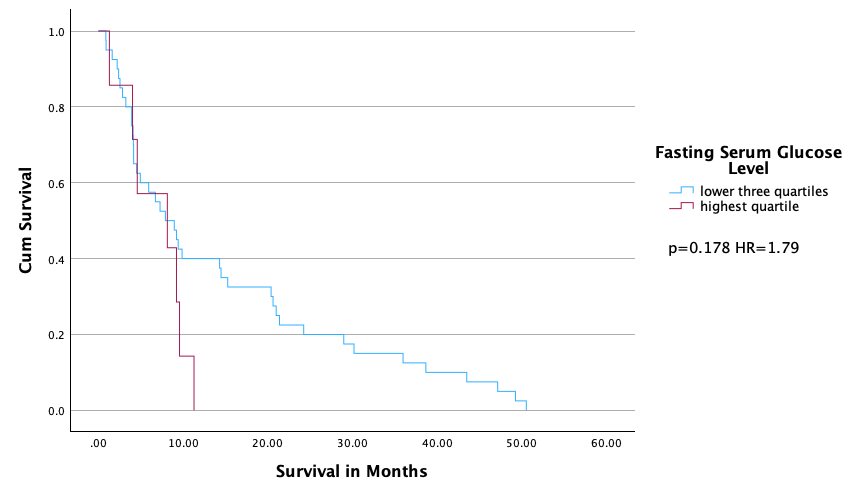


Figure 4B: Survival curves for patients without DM with fasting serum glucose level in highest and lowest quartile


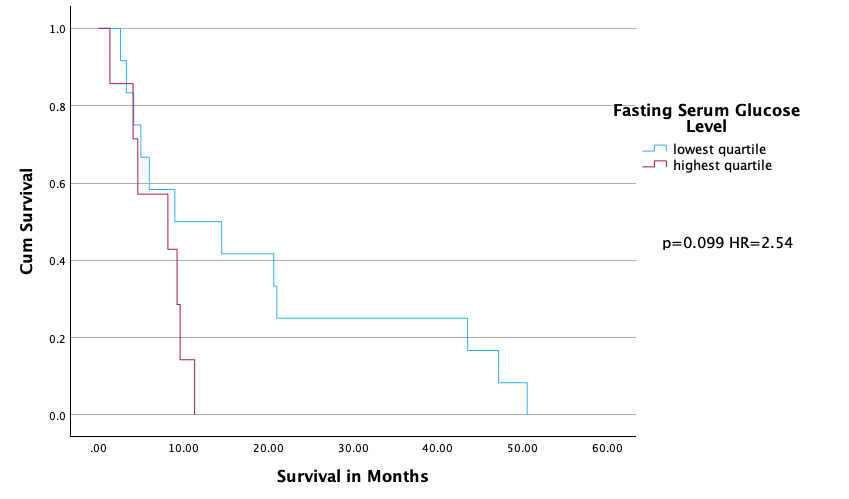


Figure 5A: Survival curves for patients without DM with maximum serum glucose level in highest and lower three quartiles


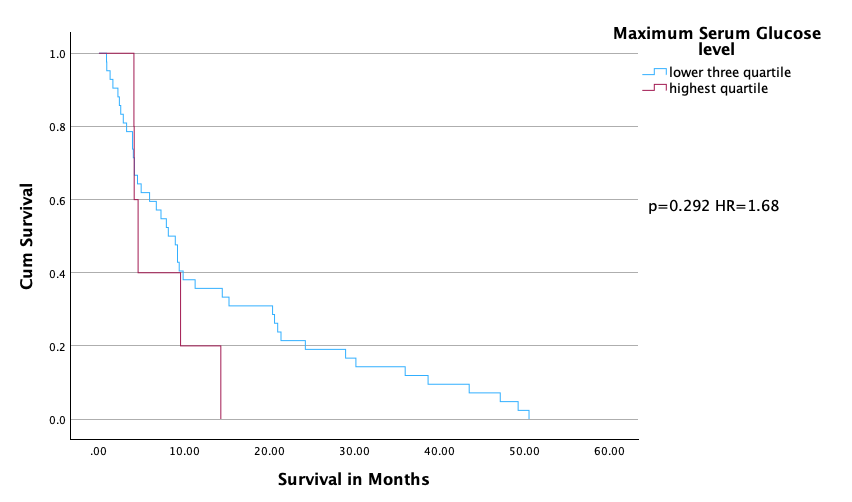


Figure 5B: Survival curves for patients without DM with maximum serum glucose level in highest and lowest quartiles


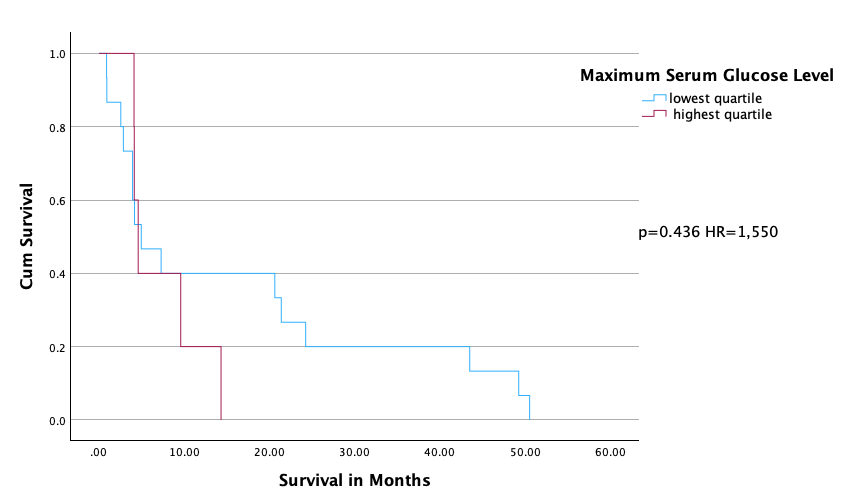


|  |
| --- |

Figure 6: Survival curve representing potential effects of antidiabetic treatment, patients with and without (oral +/- insulin) treatment of DM


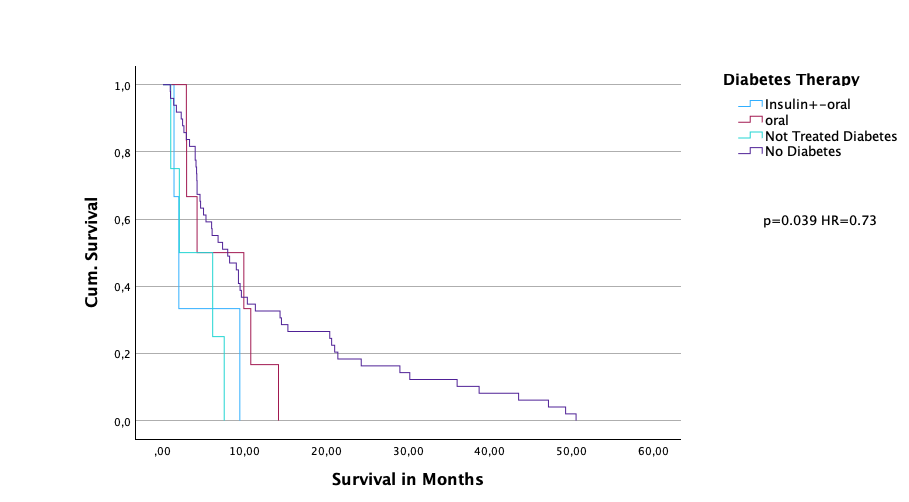


Table 1: Univariate Survival analysis

| **Characteristic** | **Median OS (95%CI)** | | ***P*** |
| --- | --- | --- | --- |
| Age |  | | HR: 1.96, p=0.026 |
| < 70 years | 7.92 (5.56-10.28) | |  |
| ≥ 70 years | 4.14 (3.97-4.31) | |  |
| KPS |  | | HR: 1.001, p=0.139 |
| ≥ 70 | 8.97 (2.88-15.06) | |  |
| < 70 | 6.08 (2.49-9.67) | |  |
| No. of brain metastases |  | | HR: 1.062, p = 0.823 |
| ≥ 3 | 6.08 (1.26-10.89) | |  |
| < 3 | 6.74 (4.06-9.42) | |  |
| Tumor type |  | | HR: 1.005, p=0.944 |
| NSCLC | 6.74 (0.685-12.80) | |  |
| SCLC | 6.08 (3.206-8.954) | |  |
| Breast cancer | 7.92 (6.817-9.023) | |  |
| Melanoma | 9.23 (2.85-15.61) | |  |
| RCC | 2.0 | |  |
| other | 4.04 (2.02-6.06) | |  |
| Radiotherapy technique |  | | HR: 1.446, p=0.319 |
| SRT | 8.97 (4.64-13.29) | |  |
| WBRT/+SRT | 6.08 (3.064-9.09) | |  |
| Corticosteroid Use |  | | HR: 1.142, p=0.611 |
| Yes | 4.17 (1.78-6.56) | |  |
| No | 7.92 (5.64-10.2) | |  |
| Gender | |  | HR: 1.411, p=0.200 |
| Female | | 7.9(3.87-11.94) |  |
| Male | | 5.95(3.33-8.57) |  |
| PAD/CHD | |  | HR: 0.800, p=0.670 |
| Yes | | 1.94(0.00-22.00) |  |
| No | | 6.74(4.29-9.19) |  |
| Hypercholesterolemia | |  | HR: 1.831, p=0.250 |
| Yes | | 4.11(1.98-6.23) |  |
| No | | 7.29(4.97-9.62) |  |
| Smoking | |  | HR: 1.14, p=0.624 |
| Yes | | 6.74(4.737-8.743 |  |
| No | | 5.95(0.48-11.41) |  |
| Arterial Hypertension | |  | HR: 1.11, p=0.688 |
| Yes | | 6.01(2.82-9.19) |  |
| No | | 7.2(2.27-12.31) |  |

**Supplement to article:**

**Hyperglycemia is associated with poor survival in patients with brain metastases treated with radiotherapy**

Soniya Poudyal^1,5*^, Friederike Rothe^1,5*^, Seong Jeong^1,5^, Nils Gleim^1,5^, Peter Hambsch^1,5^, Franziska Nägler^1,5^, Kirsten Papsdorf^1,5^, Thomas Kuhnt^1,5^, Alonso Barrantes-Freer^3^, Erdem Güresir^4^, Sabine Klagges^2^, Nils Henrik Nicolay^1,5^, Clemens Seidel^1,5^

**Corresponding author:**

**Clemens Seidel, ORCID ID 0000-0001-8178-5089**

**clemens.seidel@medizin.uni-leipzig.de**

**Klinik für Strahlentherapie und Radioonkologie, Universitätsklinikum Leipzig, Germany**
